# 

**Published:** 1842-01-01

**Authors:** 


### INDEX.

Abscess of lungs, cures of 92, 110; in the pelvis, after parturition, 223; of iliac fossæ, 432; Tickner's case, 452; behind pharynx, 169.
Acetum colchici, preparation of, 120.
" cantharides, " " 120.
Alcohol in the brain, case of, 289.
Amadou, use of, 38.
Amputation at knee joint, 136; hip joint, 205, 207; modification of flap operation, 508; mortality of, 561, 628; of leg, new mode, 623.
Amygdalitis, Chomel on, 364.
Aneurism, crural, cured by ice, 16; inguinal, 225; variety of false, 313; femoral, 604; iliac, 699.
Anchylosis of lower maxilla with temporal bones, 76.
Andral, on changes of the blood, 413.
Animals, vital action in, 465.
Angina pharyngea, 533.
Anhæmia, Channing's notes on, 669.
Aphthæ, parasites in, 572.
Aphonia, nervous, 589.
Apoplexy, 405, 513.
Arachnitis, Dr. Graves' case, 139.
Arsenic, test for, 528.
Artificial anus, 538.
Asphyxia, from drowning, 45; dissipated by snuff, 303; flagellation in, 647.
Ash, Dr. J. W., case by, 243.
Acites, treatment, 209.
Atresia vaginæ, cases of, 1, 68.
Atmosphere, constitution of, 77.
Auscultation in pregnancy, 634.
Axillary artery, obliteration of, 584,

Barton, Dr. J. R. operation by, 17, 33, 87.
Bauduy, Dr. P. J., case by, 417.
Bartlett, Dr., case by, 620; work on fever, 693.
Beck, Dr. J. B., sketch of American medicine by, 231.
Bell, Sir Charles, sketch of, 54.
Benzoic acid in urinary affections, 343, 574
Billings, work by, 214.
Blood, change of in disease, 413; buffy coat of, 717.
Bladder, paralysis of, 810; catarrh of, 282, 540.
Brinton, Dr. J. B., case by, 340.
Brain, softening of, 351.
Broussais, erection of statue to, 393.
Bright's disease, 401.
Bromine, medical properties of, 712.
Burns, creasote in, 302; treacle for, 397; new remedy for, 732.
Cancer of uterus, 152; superficial, 586; of liver, 721; of stomach, 723, 778.
Callus, cases of excision of, 205.
Catarrh of bladder, 282, 540.
Cantharides, preservation of, 363; adulterations of, 632.
Copaiva, administration of, 440.
Capital operations, statistics of, 483.
Cartilages of ribs, injury of, 512.
Cœcum, inflammation of, 541.
Catalepsy, Durand's case, 548.
Calculus, 683.
Cataract, sub-conjunctival operation for, 720.
Chapman, Dr. Nathaniel, sketch of, 322.
Chloride of zinc, as an escharotic, 411.
Chorea, caused by bad living, 608.
Clinical reports, 10, 23, 35, 38, 71, 134, 167, 177, 182, 201, 209, 234, 308, 341, 369, 370, 388, 426, 437, 452, 506, 515, 567, 817.
Clavicle, dislocation of, 63.
Climate of the U. S., 197.
Clinkers, a new remedy, 267.
Clymer, Dr., on mortality of amputations, 561.
Coates, Dr. R., on iodine, 65; contributions to surgery by, 129, 161, 262, 273, 305, 337, 449.
Connection between food and mortality, 96.
College of physicians and surgeons, 147, 168, 183, 497, 508, 725,
Constipation, 294.
Corneous excrescences of leg, 481.
Cornea, affection of in nurses, 544.
Counter-irritants, 573.
Corpora lutea, 584.
Corrosive sublimate, antidote, 641; poisoning with, 749.
Croup, Dr. Morris on, 97, 113; in adults 303; emetics in, 480.
Croton oil, external use of, 535.
Creasote, tincture of, 702.
Cryptogamia at roots of hairs, 720.
Cyanosis, 705.
Cysts in the neck, 591; in eyelids, 633; milky, 73.

Dermatalgia, rheumatic, 123.
Deformed callus, 433.
Delirium after acute meningitis, 534.
Deafness cured by morphia, 703.
Dewees, Dr. W. P., eulogium on, 755.
Diffusion, Dr. C. B. Voight on, 81.
Dislocations, of clavicle, 63; thumb, 96, of hip joint, 160, 585; 1st and 2d cervicle vertebræ, 256; of tibia backwards, 532; forwards, 543; partial outwards, 543.
Diarrhoea, treated by nitrate of silver, 264; by chloride of iodium, 301.
Diabetes mellitus 369.
Digestion, 400; chemical phenomena of, 461.
Discolouration from nitrate of silver, 703.
Diseases that never coexist, 777.
Druitt's surgery, 505.
Dropsy, O'Bierne on, 807.
Dunglison, Dr. R., work on practice by, 145; introductory by, 773.
Dysmenorrhœa, 464.

Eczema, 617.
Effects of a fruit and saccharine diet, 479.
Electricity in the human body, 115.
Elkinton, Dr. John A., case by, 514.
Elbow joint, excision of, 609: resection of, 588, 594.
Empyema, cases of, 598, 730.
Emphysema, new cure for, 736.
Epistaxis, means to arrest, 712.
Ergot, origin of, 62; use of in uterine hæmorrhage, 536; properties of, 580; use of in case of a hydatid mole, 581; in paraplegia, 650.
Erysipelas, 590.
Exophthalmia, 718.
Eyes, hysterical affection of, 548.

Fever, typhus, 26, 349, 633, 693, 812; typhoid, (latent,) 243, 620; remittent, 248.
Ferruginous bread, 300.
Fissure ani, rhatany in, 267.
Fibre, Dr. Barry on, 523, 581.
Flatus, death from, 183.
Fœtus, size of, 32; retention in utero, 689; independency of its circulation, 651.
Forry, Dr. Samuel, work of, 197.
Forceps, galvanic, 445.
Foreign bodies in ear and esophagus, 613.
Folts, Dr. D. V., case by, 801.
Fractures, effect of venereal disease or habits upon, 60; of both bones of leg, 308; Griffin's case, 317; impacted, 363; of neck of femur, 490; of radius, 538; of femur, 587; Dr. Tripler's case, 618; of forearm, 703; tables of, 704; of leg, treated by the starch bandage, 763, 802; of spine and sternum, 775.

Gangrenous affections of the puerperal state, 186.
Gerhard, Dr., on ascites, 209; diseases of chest, 217; of heart and aorta, 258.
Gelatin, nutritive properties of, 298.
Generation, spontaneous, 400.
Gibbon, Dr. Q., case by, 769.
Glossitis, case by Dr. Graves, 139.
Glanders in man, 174.
Gleet, sesquichloride of iron in, 541.
Gonorrhœa, nitrate of silver in, 138, 142.
Gouty concretions, 191.
Graves, Dr., on abscess of lungs, &c., 92, 110; arachnitis, 138; glossitis, 139; disease of heart, 357.
Green, Dr. N. T., case by, 68.

Hamilton, Dr. F. H., introductory by, 21.
Hall, Dr. M., on setons, 89; apoplexy, 405; lectures, 758.
Harris, Dr. Wm., on the uterine supporter, 194.
Hallucination and epilepsy, 285.
Harris, Dr. Thos., sketch of, 355; case by, 393.
Hay fever, 430.
Harris, Dr. C. H., cases by, 481, 785.
Hernia, strangulated, 32; belladonna in, 631; strangulated ventral, 719; radical cure of, 547; umbilical, 581; intraparietal, 717.
Hæmorrhage, after lithotomy, 32, 304; from polypus uteri, 442; tendency to, 472.
Hepatic congestion, 238.
Heart, diseases of, 258, 357; aneurism of, 287; hypertrophy of, 321; softening of, 366; malformation of, 705.
Hæmoptysis, nitrate of potassa in, 556.
Hemiplegia, facial, 578.
Hæmorrhoids, black pitch in, 635.
Hæmaturia, 812.
Hiccup cured by quinine, 121.
Homœopathy exposed, 122; Holmes on, 265.
Hodge, Dr. H. L., eulogium on Dr. Dewees, 755.
Hospitals of Italy, 782, 792.
Human body, preservation of, 513.
Hyper-operating, 44.
Hydrophobia, 237.
Hydropathy, 280, 360, 551, 766.
Hymen, Velpeau on the, 352.
Hysteria in a man, 548; a girl, 760.
Hydrencephalocele, 555.
Hydatids in bones of pelvis, 698.

Icterus, 751.
Insane, report of Pennsylvania Hospital for the, 218.
Influenza, Dr. F. C. Stewart on, 353.
Injection of air into veins, 540.
Intestinal canal, partial obliteration of, 737.
Infants, management of, 766.

Iodide of potassium in scrofula, 10; in large doses, 48; in ophthalmic disease, 241; in acute hydrocephalus, 695.
Iodine, Dr. R. Coates on, 65; in hydrocele, 142; in dropsy after scarlatina, 651; tendency of, to produce inflammation of joints, 527.
Ipecacuanha as a counter-irritant, 443.
Iron, hydrated peroxide of, 295.

Johnston, Dr. W. Poyntell, on gunshot wounds, 3, 49.
Johnston, Dr. Wm. P., case by, 401.
Jackson, Dr. S., introductory by, 742.

Kiestine, Kane on, 455.

Laryngitis, 158.
Labour, new mode of accelerating, 397; artificial premature, 496.
Larynx, external fistulæ of, 538.
Larrey, the late Baron, 629.
Leeches, parasitic, 176.
Lewis, Dr. S., case by, 289.
Lead, poisoning by, 749.
Lithotomy, lateral operation, 31, 463.
Liston's Practical Surgery, 200; Elements of, 627.
Lithotrity in a child, 236.
Lithotripsy, 817.
Light evolved from human body, 317.
Liebig's Animal Chemistry, 614; on cyanide of potassium, 653; refutation of his views on respiration, 734.
Lungs, minute structure of, 711, 762.
Lumbar abscess, 452.
Lyons, inundation of, 78.

Marsh, Dr. R. C., case by, 1.
Mamma, milky cyst in, 73.
Malpractice, Cortlandville case of, 149.
Masturbation, means of detecting, 760.
M'Clintock, Prof., introductory by, 312.
Menstruation, physiology of, 80.
Mesenteric glandular affections, 317.
Medical reform, 507.
Meigs, Dr. C. D., on puerperal fever, 529; introductory, 788.
Meigs, Dr. J. F., case by, 545.
Membrane, basement, 576.
Mettauer on diospyros Virginiana, 665.
Medical profession, difficulties of, 686.
Mercurial ointment, preparation of, 719.
Mesmerism, 725.
Milk, extemporaneous production of, 535, 748.
Miller, Dr. Thos., case by, 689.
Mitchell, Dr. J. K., on mesmerism, 725; introductory, 813.
Morris, Dr. C., on croup, 97, 113.
Musk, 541.
Mütter, Dr. T. D., introductory, 786.

Nails in fractured limbs, 12.
Nasal fosaæ, diseases of, 652.
Nervous affection following parturition, 157.
Nerves, regeneration of, 190; influence of a muscular tissue, 512; conversion into fat, 653.
Neill, Dr. J., case by, 321.
Nephritic congestion, 386.
Necrosis of femur, 545.
Neuralgia of tri-facial nerve, 673.
Nipple, cure for sore, 439, 776; chapped, 704.
Nitrate of silver, preservation of, 718.

Obstetric Catechism, Warrington's, 230.
Onixis, 768.
Ophthalmia, gonorrhœal, treatment, 287.
Orfila on absorption, 752.
Organized beings, chemistry of, 745.
Ovarian tumour, periodical, 625.
Ox gall, value of, 571.
Oxide of zinc in eczema, &c., 668.

Pain, during operations, 38; in tibia relieved by incision, 189.
Pancoast, Dr., case of amputation at knee joint, 136; of excision of elbow joint, 609.
Paralysis of seventh pair of nerves, 204; of muscles of face, 284; of bladder, 810.
Paraphymosis, treatment of, 237.
Parrish, Dr. I., on iodide of potassium, 241.
Parotid gland, congenital enlargement of, 785.
Paris, letter from, 642; sanatory condition of, 750.
Peace, Dr. E. case of inguinal aneurism by, 265; ligature of primitive iliac by, 645.
Peritonitis, 655.
Pennock, Dr. C. W., edition of Dr. Hope on the heart, 664.
Phthisis, incipient, treated by emetics, 206; ætiology of, 439; in man and animals, 684, 400; influence of tobacco on, 702; sputa in, 735.
Philadelphia College of Pharmacy, 228.
Pharmacopœia of U. S., 277.
Placenta, management of, 125.
Polypi in rectum of children, 281.
Poisoning, by corrosive sublimate, 749; by lead, 749; by binoxolate of potassia, 751.
Post-mortem examination of an aged man, 640.
Prolapsus of vagina and bladder, 171.
Pregnancy, case of, without the usual signs, 444; anomalous, 770.
Professional incomes in London, 601.
Prostate, enlarged, 634; Warner on, 682.

Puerperal convulsions, Dr. J. B. Brinton's case, 340.
Puerperal fever, 497, 529, 657.
Purgatives, use of drastic, 416.

Quinia in the blood and urine, 720.

Randolph, Dr. R. C., case by, 385.
Randolph, Dr. J., dinner to, 537.
Revaccination in Prussian army, 367.
Reunion of two fingers separated from the hand, 637.
Respirators, 687.
Rheumatism, acute articular, 140; aconite plaster in, 396; acute, Dr. Hope on, 271; use of magnet in, 592.
Rhubarb to ulcerated surfaces, 687.

Salivary glands, extirpation of, 537.
Sale, Dr., case by, 737.
Scrofula, walnut leaf tea in, 122; nature and treatment of, 549.
Scarlatina, 253.
Seatons, Dr. M. Hall on, 89.
Sharpless, Dr. John T., case by, 513.
Signs of live and still birth, 245.
Skin, functions of, 191, 303.
Small-pox, identity with cow-pox, 476.
Spinal distortions, 496.
Spinal irritation after intermittents, 590.
Spleen, anatomy of, 601.
Splint, Nottingham's, 648.
Stewardson, Dr. T., on yellow fever, 56; on remittent fever, 248.
Stomach, perforations of, 76; Dr. Fondey's case of enlarged, 595; cramp in, 302.
Strabismus, results of operations for, 141, 583; Bolton's instruments for, 567; Velpeau on, 675.
Stewart, Dr. F. C., on epidemic influenza, 353; case by, 817.
Surgical practice of Hotel Dieu, 709.
Surgery, contributions to, 129, 161, 262, 273, 305, 337, 449.
Sudden death, pathology and cases of, 378.
Surgical instruments, gilding of, 548.
Sweat, organs for secretion of, 632.

Tannin, an antidote to strychnia, 535.
Tarsal bones, disease of, 522.
Tape-worm, 269.
Teeth, extraction of, 25; Harris' work on, 54; fatal hæmorrhage from, 344; structure of, 764.
Tenotomy, 107.
Tetanus, cases of, 299, 417, 556, 769.
Therapeutics, Symonds on, 795.
Tickner, Dr., letter of, 87.
Tic-douloureux, croton oil in, 220.
Tonsils, excision of, 668.
Trismus neonatorum, cases of, 124, 347.
Tracheotomy in croup, 137.
Trachea, structure of, 207; foreign body in, 463.
Trichiasis, 442.
Transfusion, 748.
Tubercular disease, cases of, 114, 166; comparative frequency of, 477.
Tubercle, vascularity of, 189.
Tumours in cauda equina, 361; in neck, 375.
Tucker, Dr. D. H., case by, 452.
Twins, Dr. Skipton's case of, 573: Dr. Jameson's, 697.

Ultimate secreting structure, 557.
Upper Maxilla, excision of, 603.
Urea secreted by peritoneum, 511.
Urethroplasty, 378.
Urethra, stricture of, 445, 763; circular stricture of, 462; obliteration of, 463.
Urine, composition of, in disease, and in pregnant women, 287, 832.
Urinary calculi, solution of, 463.
Urino-genital organs, 777.
Uterine supporter, Dr. W. Harris on, 194.
Uterine polypi, extirpation of, 236.
Uterus, cauterization of neck of, 464; recovery after extirpation of, 572; rupture of, by ergot, 583; apthæ of neck of, 763.

Vaccine virus, durability of, 549.
Vaccination, 735.
Vagina, prolapsus of, 510.
Vaginitis, 748.
Varicose veins, treatment of, 41, 282.
Velpeau, on varicose veins, 282; on the hymen, 352; mode of administering cubebs, &c. 377, on fracture of radius, 538; cysts in eyelids, 633: strabismus, 675.
Venous hemorrhage from an abscess, 410.
Voight, Dr. C. B., on diffusion, 81.

Wallace, Dr. J. M., antidote to corrosive sublimate, 641.
Warty excrescences, blistering for, 239.
West, Dr., case by, 705.
Wilson, Dr. M. W., on puerperal fever, 657.
Wild cherry bark, syrup of, mode of preparing, 313.
Wood, Dr. Geo. B., introductory by, 737.
Wounds, gun-shot, 3, 49; of lungs, 414; of antrum highmorianum, 687.
Wry neck, operations for, 14.

### TO SUBSCRIBERS.

The MEDICAL EXAMINER will hereafter be edited and conducted by Dr. M. CLYMER. It will be published once in two weeks, on every alternate Saturday, commencing with the 21st of January, at the present subscription price. Each number will contain more than twice the amount of matter now given in one number, thus reducing the postage one half. The cover for advertisements will be retained, but will form part of the sheet containing the body of the work, thereby effecting a further reduction in the postage. The first number of the new series will be sent to the present subscribers to the Examiner. Such as are desirous of being retained on the subscription list, are requested to remit the subscription in advance, after the receipt of the first number.

A portion of each number will be devoted to the publication of the Clinical Lectures, and Hospital Reports of this and other cities of the Union; and a regular series of lectures by the prominent Clinical Professors in Paris, will be given, reliable arrangements having been made to that effect. A large list of valuable contributors has been secured, whose active collaboration may be depended on.

Subscribers who are in arrears for past years, are earnestly requested at once to liquidate them. The editors feel grateful to the numerous body of subscribers who have punctually forwarded their subscriptions, and they trust that the present appeal will be responded to by those whose accounts are unsettled.

